# Evaluation of the inhibitory potentials of selected compounds from *Costus spicatus* (Jacq.) rhizome towards enzymes associated with insulin resistance in polycystic ovarian syndrome: an in silico study

**DOI:** 10.1186/s43141-021-00276-2

**Published:** 2021-11-23

**Authors:** Fehintoluwa Joy Femi-Olabisi, Ahmed Adebayo Ishola, Opeyemi Faokunla, Anthonia Oluyemi Agboola, Benjamin Ayodipupo Babalola

**Affiliations:** 1 Biochemistry Unit, Department of Biological Sciences, Mountain Top University, Prayer city, Nigeria; 2 Central Research Laboratory, 132B University road, Tanke, Ilorin, Kwara State Nigeria; 3 Department of Biochemistry, Federal University Lokoja, Lokoja, Kogi State Nigeria; 4 Biochemistry Unit, Department of Biological Sciences, Wesley University, Ondo, Nigeria

**Keywords:** Polycystic ovary syndrome (PCOS), PEPCK, α-amylase, β-glucosidase, FBPase, GC-MS

## Abstract

**Background:**

Polycystic ovary syndrome (PCOS) is a chronic endocrine disorder prevalent in premenopausal women and is characterized by a range of physiological and biochemical abnormalities which may include reproductive, endocrine, and metabolic alterations such as insulin resistance. Insulin resistance is the hallmark of PCOS as it predisposes the affected subjects to a higher risk of impaired glucose tolerance and type 2 diabetes mellitus (T2DM). In this study, the inhibitory activities of phytosterols and saccharides from aqueous extract of *Costus spicatus* rhizome were investigated against phosphoenolpyruvate carboxykinase (PEPCK), α-amylase, β-glucosidase, and fructose 1,6-biphosphatase (FBPase) in silico as potential novel therapeutic targets for T2DM-associated-PCOS. Phytochemical constituents of the plant were determined using gas chromatography-mass spectrophotometry (GC-MS), while molecular docking of the compounds with PEPCK, α-amylase, β-glucosidase, and FBPase was conducted using Vina. Thereafter, the binding modes were determined using Discovery Studio Visualizer, 2020.

**Results:**

GCMS analysis of an aqueous extract of *Costus spicatus* rhizome revealed the presence of three compounds with a higher binding affinity for all enzymes studied compared to metformin. The compounds also interacted with key amino acid residues crucial to the enzyme’s activities. This study identified Lyxo-d-manno-nononic-1,4-lactone as potential multi-target inhibitors of PEPCK, α-amylase, β-glucosidase, and FBPase with reasonable pharmacokinetic properties and no significant toxicity.

**Conclusion:**

These compounds can be explored as potential therapeutic agents for the management of insulin resistance in PCOS, subject to further experimental validation.

## Background

Advances in drug discovery have been possible with the advent of bio-computational methods, artificial intelligence, and machine learning which accelerates drug discovery, lead optimization, drug development, and design [1]. In biomedicine, in silico architecture is used to streamline and accelerate hit detection and hit-to-lead optimization processes using computer-aided techniques in the drug discovery process [2, 3]. Several computational techniques have been proposed to recognize and select therapeutically relevant targets, study the molecular basis of drug-receptor complexes interactions, and estimate binding free energy between a ligand and receptor in order to rationalize and improve the productivity, speed, and cost-efficiency of the drug discovery process [4, 5].

Polycystic ovarian syndrome (PCOS) which is characterized by anovulation, oligomenorrhea, amenorrhea, hyperandrogenism, and polycystic ovaries, often exhibits non-reproductive metabolic abnormalities such as obesity, hyperinsulinemia, insulin resistance, and dyslipidemia with a risk of T2DM [6–8]. Women with PCOS are predisposed to a higher risk of impaired glucose tolerance and T2DM as insulin resistance is a hallmark of the disorder [9]. In diabetic patients, an altered rate of gluconeogenesis is responsible for increased hepatic glucose output (HGO) and therefore sustained hyperglycemia is observed in both insulin-dependent DM and non-insulin-dependent DM [10]. Expression of the gene for cytosolic phospho*enol*pyruvate carboxykinase (PEPCK-C) in pancreatic α-cells is induced during diabetes, both in animals and in human patients [11]. *α*-Amylase is responsible for postprandial glucose levels and has become a therapeutic target for the management of T2DM through inhibition to decrease the reabsorption of glucose in the intestine and postprandial blood glucose levels [12]. β-Glucosidase is an enzyme involved in complex carbohydrate dissociation in the small intestine that plays its role to cleave glycosidic bonds, resulting in glucose release from the non-reducing end of an oligo- or poly-saccharide chain involved in glycoprotein biosynthesis [13]; its inhibitors were reported to be of interest owing to their therapeutic potential in diabetes treatment [14]. Also, the inhibitors of fructose-1,6-Bisphophatase—a regulatory enzyme of gluconeogenesis that catalyze the penultimate step of the pathway and controls glucose production from all non-carbohydrate precursors—was recently reported to inhibit endogenous glucose production towards the management of elevated glucose levels [15]. Medicinal plants encompass a rich source of active compounds that have anti-diabetic properties, and *Costus spicatus* (Insulin plant) rhizome has been used in folkloric medicine to treat diabetes mellitus and other associated pathological manifestations [16, 17]. The current therapeutic approach for treating PCOS involves the use of insulin sensitizers such as metformin which is rather costly and may be unaffordable. In this study, phyto-compounds from aqueous extract of *Costus spicatus* rhizome were investigated for their inhibitory activities against PEPCK, α-amylase, β-glucosidase, and fructose-1,6-bisphosphatase in silico as potential novel therapeutic targets for insulin resistance treatment associated with PCOS.

## Methods

### Plant material

Fresh rhizomes of *Costus Spicatus* were collected within the premises of Mountain Top University. The plant was identified at the Botany Department of the University of Lagos, where a Voucher Specimen Number 8571 was prepared and deposited at the Department’s herbarium.

### Plant preparation

A known weight (1 kg) of *Costus Spicatus* rhizome was washed, air-dried, and pulverized in a blender. The powdered material (1 kg) was extracted in 4 L of distilled water for 48 h and filtered with Whatman No. 1 filter paper. The filtrate was lyophilized to give a yield of 47.57 g which corresponds to 15.85%. The concentrate was then stored in a refrigerator at −4°C.

### Gas chromatography mass spectrophotometry

The GC-MS analysis was carried out using a Hewlett Packard Gas Chromatograph (Model 6890 series) fitted with a Hewlett Packard 7683 series flame ionization detector and a 250°C MS transfer line temperature injector.

### Protein preparation

The crystal structures of phosphoenolpyruvate carboxykinase, α-amylase, β-glucosidase, and fructose-1,6, biphosphatase with PDB IDs, 2GMV, 3BAW, 2JFE, and 5Q09, respectively were obtained from protein databank (www.rcsb.org). All the crystal structures were prepared individually by removing existing ligands and water molecules, while missing hydrogen atoms were added using Autodock v4.2 program, Scripps Research Institute. Thereafter, non-polar hydrogens were merged while polar hydrogen where added to each enzyme. The process was repeated for each protein and subsequently saved into pdbqt format in preparation for molecular docking.

### Ligand preparation

The SDF structures of metformin and the test ligands were retrieved from the PubChem database (www.pubchem.ncbi.nlm.nih.gov). The compounds were converted to mol2 chemical format using Open babel [18]. Polar hydrogens were added while non-polar hydrogens were merged with the carbons, and the internal degrees of freedom and torsions were set. The ligand molecules were further converted to the dockable pdbqt format using Autodock tools.

### Molecular docking

Docking of the ligands to various protein targets and determination of binding affinities were carried out using Vina [19]. Pdbqt format of the receptors, as well as those of the ligands, was dragged into their respective columns and the software was run. For PEPCK, the grid box was centered at *X* = 26.28, *Y* = −2.60, and *Z* = 21.86, and the dimension of the grid box was set at 57.23 × 84.24 × 21.86, for α-amylase, the grid box was centered at *X* = 7.35, *Y* = −6.29, and Z = −17.72, and the dimension of the grid box was set at 65.48 × 80.79 × 66.49. For ß-galactosidase, the grid box was centered at *X* = 35.70, *Y* = 53.42, and *Z* = 35.67, and the dimension of the grid box was set at 66.72 × 59.81 × 71.91. For FBPase, the grid box was centered at *X* = 22.26, *Y* = 22.28, and *Z* = −3.54 and the dimension of the grid box was set at 75.27 × 63.42 × 65.56. The binding affinities of compounds for the three protein targets were recorded. The compounds were subsequently ranked by their affinity scores. Molecular interactions between the receptors and compounds with most remarkable binding affinities were viewed with Discovery Studio Visualizer, BIOVIA, 2020.

### ADMET studies

The toxicity risks of metformin were predicted based on its ADMET profile. The ADMET (absorption, distribution, metabolism, excretion, and toxicity) studies were predicted using pkCSM tool (http://biosig.unimelb.edu.au/pkcsm/prediction) [20]. The SMILE molecular structures of the compounds were obtained from PubChem (https://pubchem.ncbi.nlm.nih.gov).

## Results

The qualitative phytochemical analysis of an aqueous extract of *Costus spicatus* rhizome extract revealed the presence of alkaloids, flavonoids, carbohydrates, and proteins which are important in disease prevention and health preservation. The GC-MS chromatogram of aqueous extracts of *Costus spicatus* rhizome showed the spikes of a total of thirteen compounds identified as shown in Table 1. Molecular docking revealed the binding of the standard (metformin) and other ligands to phosphoenolpyruvate carboxykinase, α-amylase, β-glucosidase, and fructose-1,6, biphosphatase (Table 1). Compounds with the highest binding affinity to the corresponding phosphoenolpyruvate carboxykinase, α-amylase, β-glucosidase, and fructose-1,6, biphosphatase were indicated in bold values. d-Lyxo-d-manno-nononic-1,4-lactone, oleic acid, and cyclohexane, 1,1’-(2-methyl-1,3 propanediyl)bis- had a binding affinity of −8.3, −8.1, and −7.4 Kcal/mol, respectively, for phosphoenol pyruvate carboxykinase compared to metformin’s −7.3 Kcal/mol. Also, d-Lyxo-d-manno-nononic-1,4-lactone, oleic acid, and cyclohexane, 1,1’-(2-methyl-1,3 propanediyl)bis- had a binding affinity of −7.9, −7.8, and −8.1 Kcal/mol for α-amylase compared to metformin’s −7.6 Kcal/mol. Similar results were obtained for the aforementioned compounds using ß-galactosidase and fructose 1,6 biphosphate. d-Lyxo-d-manno-nononic-1,4-lactone, oleic acid, and cyclohexane, 1,1’-(2-methyl-1,3 propanediyl)bis- had a binding affinity of −8.5, −7.9, −8.8 Kcal/mol, and −8.6, −7.7, and −7.9 Kcal/mol compared to metformin’s −7.7 and −7.4 kcal/mol, respectively, for the two enzymes.

**Table 1:** Binding affinity of ligands to phosphoenolpyruvate carboxykinase, α-amylase, β-glucosidase, and fructose-1,6, biphosphatase

	Compounds	Binding affinity (Kcal/mol)			
		PEPCK	α-amylase	β-glucosidase	fructose-1,6, biphosphatase
R	Metformin	**−7.3**	**−7.6**	**−7.7**	**−7.4**
1	Methyl 6-O-[1-methylpropyl]-beta-d-galactopyranoside	**−**7.1	**−**7.2	**−**7.3	**−**6.8
2	O-Decylhydroxylamine	**−**6.2	**−**5.9	**−**7.1	**−**5.0
3	Acetic acid phenyl ester	**−**6.0	**−**5.5	**−**6.3	**−**5.9
4	Formic acid phenyl ester	**−**5.9	**−**5.4	**−**5.9	**−**5.8
5	d-Lyxo-d-manno-nononic-1,4-lactone	**−8.3**	**−7.9**	**−8.5**	**−8.6**
6	Galacto-heptulose	**−**7.0	**−**7.0	**−**7.6	**−**6.7
7	Palmitoleic acid	**−**6.8	**−**7.5	**−**7.6	**−**6.0
8	Oleic acid	**−8.1**	**−7.8**	**−7.9**	**−7.7**
9	Hexadecanoic acid, methyl ester	**−**6.8	**−**7.0	**−**7.2	**−**6.5
10	9,12-Octadecadienoic acid, methyl ester, (E,E)-	**−**7.0	**−**7.2	**−**6.7	**−**6.5
11	[1,2,4]Triazolo[1,5-a]pyrimidine-6 -carboxylic acid, 4,7-dihydro-7-imino-, ethyl ester	**−**6.7	**−**6.4	**−**6.8	**−**6.7
12	Carbonic acid, but-3-en-1-yl penta decyl ester	**−**7.2	**−**7.1	**−**7.1	**−**7.3
13	Cyclohexane, 1,1′-(2-methyl-1,3 propanediyl)bis-	**−7.4**	**−8.1**	**−8.8**	**−7.9**

**Fig. 1:**
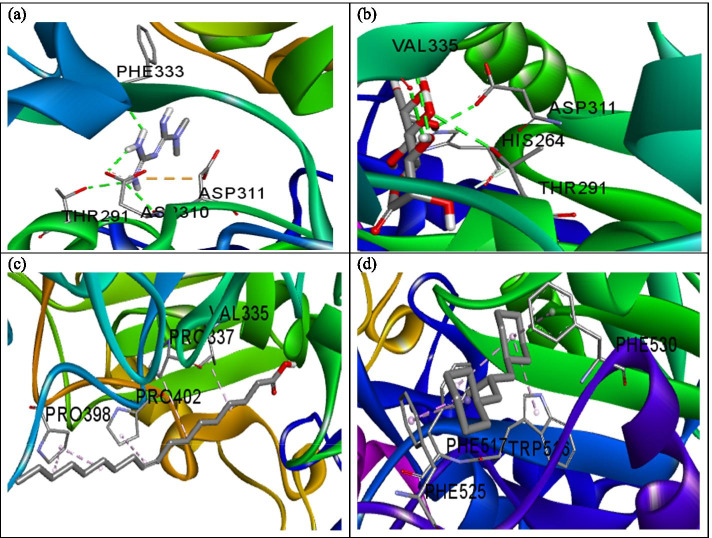
A 3D view of the interaction between **a** metformin, **b** d-Lyxo-d-manno-nononic-1,4-lactone, **c** oleic acid, and **d** Cyclohexane, 1,1’-(2-methyl-1,3 propanediyl)bis and phosphoenol pyruvate carboxykinase

A total of three hydrogen bonds with Thr291, Asp310, and Phe333 were observed in the binding of metformin to PEPCK in addition to two non-hydrogen bonds (Fig. 1a). Also, hydrogen bond was the only major mode of interaction between d-Lyxo-d-manno-nononic-1,4-lactone and PEPCK (Fig. 1b). However, hydrophobic interaction was the only means of interaction between PEPCK and oleic acid as well as cyclohexane, 1,1’-(2-methyl-1,3 propanediyl)bis- (Fig. 1c, d) (Table 2).

**Table 2:** Summary of bond formation between ligands and enzymes

	Compounds	Binding affinity (Kcal/mol)			
		PEPCK	α-amylase	β-glucosidase	Fructose-1,6, biphosphatase
R	Metformin	**H-bond** Thr291, Asp310, Phe333 **Non-H-bond** Asp310, Asp311	**H-bond** Pro332, Gly334, Arg398. **Non-H-bond** Asp402	**H-bond** Gln17, Glu165, Phe225, Tyr309 **Non-H-bond** Glu373, Trp417, Glu424, Trp425	**H-bond** Glu98, Asp118. Leu120, Asp121, Glu280 **Non-H-bond** Asp74, Glu97, Glu98
1	d-Lyxo-d-manno-nononic-1,4-lactone	**H-bond** His264, Thr291, Asp311, Val335	**H-bond** Arg267, Asn301, Gly309	**H-bond** Glu165, Tyr309, Glu345, Glu373, Trp425, Gln427	**H-bond** Glu97, Leu120, Ser123, Ser124, Tyr244, Gly246
2	Oleic acid	**Non-H-bond** Val335, Pro337, Pro398, Pro402	**Non-H-bond** Trp59, Tyr62, Ala106, Leu165	**Non-H-bond** Val168, Met172, Phe225, Ile332, Tyr309, Trp345, Trp417	**Non-H-bond** Leu56, Lys72, Leu73, Ile126
3	Cyclohexane,1,1′-(2-methyl-1,3 propanediyl)bis-	**Non-H-bond** Trp516, Phe517, Phe525	**Non-H-bond** Trp59, Tyr62	**Non-H-bond** Val171, Val227, Leu229, His250, Ala246, Phe334, Leu253, Leu327	**Non-H-bond** Tyr264, Pro265

**Fig. 2:**
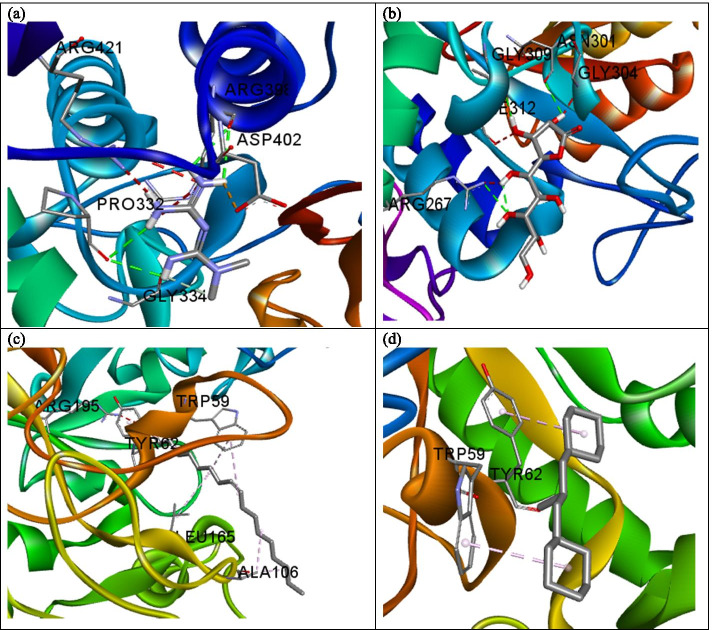
A 3D view of the interaction between **a** metformin, **b** d-Lyxo-d-manno-nononic-1,4-lactone, **c** oleic acid, and **d** Cyclohexane, 1,1’-(2-methyl-1,3 propanediyl)bis and α-amylase

**Fig. 3:**
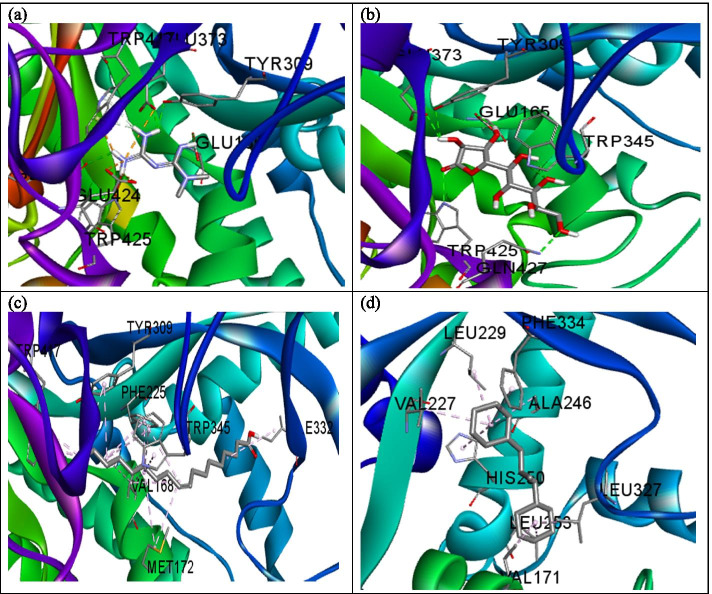
A 3D view of the interaction between **a** metformin, **b** d-Lyxo-d-manno-nononic-1,4-lactone, **c** oleic acid, and **d** Cyclohexane, 1,1’-(2-methyl-1,3 propanediyl)bis and ß-glucosidase

**Fig. 4:**
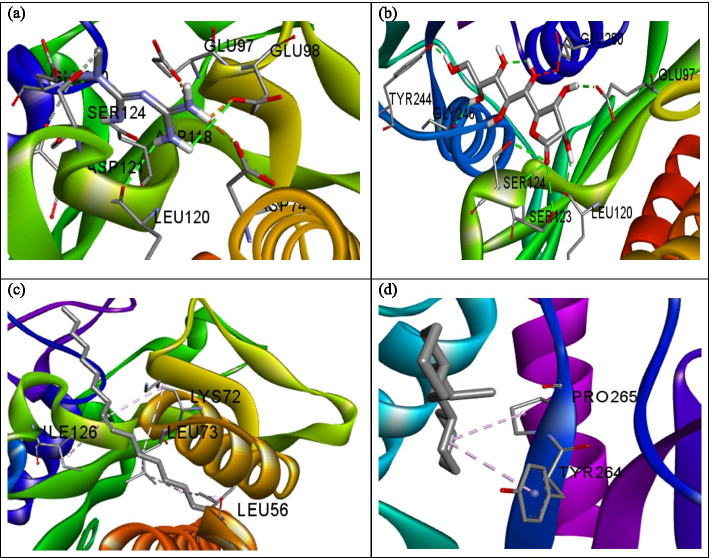
A 3D view of the interaction between **a** metformin, **b** d-Lyxo-d-manno-nononic-1,4-lactone, **c** oleic acid, and **d** Cyclohexane, 1,1’-(2-methyl-1,3 propanediyl)bis and fructose -1,6, biphosphatase

The influence of the hydrogen bond is visible in the interaction of metformin and α-amylase (Fig. 2a) where a total of three hydrogen bonds were formed. As observed with PEPCK, d-Lyxo-d-manno-nononic-1,4-lactone interacted with α-amylase solely via hydrogen bond (Fig. 2b) while the interaction of oleic acid and cyclohexane, 1,1’-(2-methyl-1,3 propanediyl)bis- followed a similar pattern with only hydrophobic interaction with the enzymes studied (Fig. 2c, d). Metformin interacted with ß-glucosidase via hydrogen bond with Gln17, Glu165, Phe225, and Tyr309 (Fig. 3a). d-Lyxo-d-manno-nononic-1,4-lactone was involved in hydrogen bond interaction with Glu165, Tyr309, Glu345, Glu373, Trp425, and Gln427 of ß-glucosidase (Fig. 3b) while oleic acid interacted with Val168, Met172, Phe225, Ile332, Tyr309, Trp345, and Trp417 of ß-glucosidase via hydrophobic interaction (Fig. 3c). Similarly, Cyclohexane,1,1’-(2-methyl-1,3 propanediyl)bis was involved in hydrophobic interaction with Val171, Val227, Leu229, His250, Ala246, Phe334, Leu253, and Leu327 of ß-glucosidase (Fig. 3d). The standard drug (metformin) interacted with FBPase via hydrogen bond with Glu98, Asp118. Leu120, Asp121, Glu280, and π-anion interaction with Asp74, Glu97, and Glu98 of FBPase (Fig. 4a). As observed for the other enzymes, d-Lyxo-d-manno-nononic-1,4-lactone was involved in solely hydrogen bond formation with Glu97, Leu120, Ser123, Ser124, Tyr244, and Gly246 of FBPase (Fig. 4b). Oleic acid elicited hydrophobic interactions with Leu56, Lys72, Leu73, and Ile126 of FBPase (Fig. 4c) while Cyclohexane,1,1’-(2-methyl-1,3 propanediyl)bis- was also involved in hydrophobic interaction with Tyr264, Pro265 of the enzyme (Fig. 4d). The ADMET properties of metformin and selected phytomolecules identified by this study are shown in Table 3. Molecules with less than 30% absorbance were considered to be poorly absorbed in the intestine [20]. Consequently, d-Lyxo-d-manno-nononic-1,4-lactone was poorly absorbed (<30%). On the contrary, Cyclohexane, 1,1’-(2-methyl-1,3 propanediyl)bis and oleic acid possess good absorption properties with a 93.62 and 91.77% value.

**Table 3:** ADMET properties of potential inhibitors of anti-insulinemic resistance targets

		**Absorption**					
		Intestinal absorption (%)	Water solubility (log mol/L)	Skin Permeability (log Kp)	P-glycoprotein substrate	P-glycoprotein I inhibitor	P-glycoprotein II inhibitor
R	Metformin	59.40	**−**2.657	**−**2.74	Yes	No	No
1	d-Lyxo-d-manno-nononic-1,4-lactone	22.72	**−**1.15	**−**0.33	Yes	No	No
2	Oleic acid	91.77	**−**5.69	**−**2.52	No	No	No
3	Cyclohexane, 1,1′-(2-methyl-1,3 propanediyl)bis	93.62	**−**7.10	**−**2.28	No	No	No
		**Distribution**					
		VDss (human) (log L/kg)	Fraction unbound (human)	BBB permeability	CNS permeability		
R	Metformin	**−**0.33	0.88	**−**0.80	**−**4.24		
1	d-Lyxo-d-manno-nononic-1,4-lactone	**−**0.03	0.85	**−**1.3	**−**4.31		
2	Oleic acid	**−**0.57	0.046	**−**0.18	**−**1.65		
3	Cyclohexane, 1,1′-(2-methyl-1,3 propanediyl)bis	0.53	0.009	0.871	**−**1.07		
		**Metabolism**					
		CYP2D6 substrate	CYP3A4 substrate	CYP1A2 inhibitor	CYP2C19 inhibitor	CYP2C9 inhibitor	CYP3A4 inhibitor
R	Metformin	No	No	No	No	No	No
1	d-Lyxo-d-manno-nononic-1,4-lactone	No	No	No	No	No	No
2	Oleic acid	Yes	Yes	Yes	No	No	No
3	Cyclohexane, 1,1′-(2-methyl-1,3 propanediyl)bis	No	Yes	Yes	No	No	No
		**Excretion**		**Toxicity**			
		Total Clearance (log ml/min/kg)	Renal OCT2 substrate	Max. tolerated dose (log mg/kg/day)	AMES toxicity	Hepatotoxicity	Skin sensitization
R	Metformin	0.1	No	0.81	Yes	No	Yes
1	d-Lyxo-d-manno-nononic-1,4-lactone	0.82	No	1.92	No	No	No
2	Oleic acid	1.88	No	**−**0.94	No	Yes	Yes
3	Cyclohexane, 1,1′-(2-methyl-1,3 propanediyl)bis	1.22	No	**−**0.51	No	No	Yes

## Discussion

Insulin resistance (IR) is prevalent in PCOS, and it can be considered an integral part of the syndrome. IR and glucose metabolism deregulation are currently reported to play a pathogenic role in the disease. IR leads to hyperinsulinemia, which increases ovarian androgen synthesis both by direct ovarian actions and by stimulating LH secretion [21]. IR also induces dyslipidemia, and women with PCOS have an increased risk of type 2 diabetes mellitus and cardiovascular disease [22]. In the present study, we carried out in silico experiments to evaluate the binding tendencies of phosphoenolpyruvate carboxykinase, β-glucosidase, fructose-1,6-bisphosphatase, and α-amylase associated with insulin resistance in polycystic ovarian syndrome to fatty acids and saccharides from the aqueous extract of *Costus spicatus* rhizome.

*Costus spicatus* has many therapeutic actions and scientifically validated documents. The qualitative phytochemical analysis of an aqueous extract of *Costus spicatus* rhizome revealed compounds of great interest. These compounds are of pharmacological importance as they possess the properties such as anti-diabetic, analgesic, antibacterial, and antifungal activity.

The more negative the value of the docking score, the higher the affinity of ligand toward the receptor [23]. d-Lyxo-d-manno-nononic-1,4-lactone, oleic acid, and cyclohexane, 1,1’-(2-methyl-1,3 propanediyl)bis- had a more negative binding affinity for the enzymes studied compared to metformin. Metformin has been reported to work by minimizing the absorption of intestinal glucose, enhancing peripheral glucose uptake, reducing the amount of fasting plasma insulin, and rising insulin sensitivity, resulting in a drop in blood glucose levels without inducing hypoglycemia [24]. The higher binding affinity of these ligands compared to metformin indicated higher inhibitory activities to the overexpression of PEPCK, α-amylase, β-glucosidase, and FBPase and thereby serve as potential novel therapeutic targets for insulin resistance treatment associated with PCOS.

The high percentage absorption observed for compounds isolated from the flower may be due to the inhibition of P-glycoprotein I and II. P-glycoprotein forms an aqueous transmembrane pore through which drugs are actively transported from the cytosol to the extracellular media [25]. Therefore, the ability of compounds to inhibit this protein is crucial to the bioavailability of drugs as increased intestinal expression of P-glycoprotein could reduce the absorption of compounds as observed in this study. Of all compounds, only d-Lyxo-d-manno-nononic-1,4-lactone, was not blood-brain barrier (BBB) and central nervous system (CNS) permeant due to its low lipophilicity as compounds with log BB less than −1.0 and log PS less than −3.0 are considered to be poorly distributed in the brain and central nervous system, respectively. This suggests that the compounds do not possess neuromodulatory ability and may effectively get to other targets.

The volume of distribution at steady state (VDss) is the volume of blood necessary to hold the compound present in the body at the concentration observed in the vascular compartment [26]. The higher the VDss value the more likely the drug is distributed in the tissue rather than plasma, results obtained showed that four lead compounds from the flower had a lower theoretical dose required for uniform distribution in the plasma.

Cytochrome P450 (CYP) enzymes are responsible for the metabolism of most clinically used drugs [27]. Out of over 50 known CYP enzymes, CYP1A2, CYP2C9, CYP2C19, CYP2D6, CYP3A4, and CYP3A5 enzymes metabolize about 90% of drugs [28]. This study shows that the three lead compounds might not inhibit most of the cytochrome enzymes. This suggests that the compounds might not cause drug-drug interaction associated with the inhibition of CYP enzymes as this may be life-threatening. Several drugs have been withdrawn from the market because metabolic inhibition by other drugs leads to life-threatening conditions [29].

None of the hit compounds is a substrate for renal OCT2 substrate, a poly-specific, bi-directional, facilitative diffusional transporter predominantly expressed on the basolateral membrane of kidney proximal tubule. Consequently, the predictive mode of elimination of the compounds was via sweat, bile, and feces.

The maximum tolerated dose provides an estimate of the toxic dose threshold of drugs in humans. As a standard, a maximum tolerated dose lower than or equal to 0.477 (log mg/kg/day) is considered low and high if more than 0.477 (log mg/kg/day). Consequently, the hit compounds identified in this study may only be administered at low doses due to their low maximum tolerated dose. This property is normal for potent drugs as ideal drug candidates are effective at low doses. AMES toxicity is widely employed to assess the mutagenic potential of compounds using bacteria. Results obtained from the AMES study suggest that the compounds might not be mutagenic and therefore may not act as carcinogens. d-Lyxo-d-manno-nononic-1,4-lactone may not be hepatotoxic and may not be sensitive to the skin as shown in the predictive toxicity test while oleic acid may be hepatotoxic and sensitive to the skin. In all, d-Lyxo-d-manno-nononic-1,4-lactone possessed reasonable pharmacokinetic properties expected for promising drugs that could further be explored using other models prior to their enlistment as a novel therapy for insulin resistance associated with PCOS.

## Conclusion

GC-MS analysis carried out on the aqueous extract of *Costus spicatus* rhizome revealed the presence of compounds such as galactoheptulose, oleic acid, methyl 6-O-[1-methylpropyl]-beta-d-galactopyranoside, and d-lyxo-d-manno-nonionic-1,4-lactone, Cyclohexane, and 1,1’-(2-methyl-1,3 propanediyl)bis. Three of these compounds; d-Lyxo-d-manno-nononic-1,4-lactone, oleic acid and Cyclohexane, 1,1’-(2-methyl-1,3 propanediyl)bis were identified as multiple inhibitors of PEPCK, α-amylase, β-glucosidase, and fructose-1,6-bisphosphatase as compared to standard drug metformin and could therefore inhibit gluconeogenesis, decrease glucose absorption, and also increase insulin sensitivity. d-Lyxo-d-manno-nononic-1,4-lactone stand out both in terms of its binding affinity for the enzymes studied and also possessed reasonable pharmacokinetic properties with no risk of toxicity and thereby qualified for further studies using different models before enlistment as a potential therapy against insulin resistance associated with PCOS.

## Data Availability

Not applicable

## Abbreviations

PCOS: Polycystic ovarian syndrome
T2DM: Type-2 diabetes mellitus
PEPCK: Phosphoenolpyruvate carboxykinase
FBPase: Fructose-1,6-bisphosphatase
GC-MS: Gas chromatography mass spectrophotometry
DM: Diabetes mellitus
HGO: Hepatic glucose output
ADMET: Absorption Distribution Metabolism Excretion and Toxicity
IR: Insulin resistance
CNS: Central nervous system
VDss: Volume of distribution at steady state
CYP: Cytochrome P_450_
